# Toward targeting inflammasomes: insights into their regulation and activation

**DOI:** 10.1038/s41422-020-0295-8

**Published:** 2020-03-09

**Authors:** Shelbi Christgen, David E. Place, Thirumala-Devi Kanneganti

**Affiliations:** Department of Immunology, St. Jude Children’s Research Hospital, Memphis, TN 38105 USA

**Keywords:** NOD-like receptors, Innate immunity, Pattern recognition receptors, Structural biology, Transcriptional regulatory elements

## Abstract

Inflammasomes are multi-component signaling complexes critical to the initiation of pyroptotic cell death in response to invading pathogens and cellular damage. A number of innate immune receptors have been reported to serve as inflammasome sensors. Activation of these sensors leads to the proteolytic activation of caspase-1, a proinflammatory caspase responsible for the cleavage of proinflammatory cytokines interleukin-1β and interleukin-18 and the effector of pyroptotic cell death, gasdermin D. Though crucial to the innate immune response to infection, dysregulation of inflammasome activation can lead to the development of inflammatory diseases, neurodegeneration, and cancer. Therefore, clinical interest in the modulation of inflammasome activation is swiftly growing. As such, it is imperative to develop a mechanistic understanding of the regulation of these complexes. In this review, we divide the regulation of inflammasome activation into three parts. We discuss the transcriptional regulation of inflammasome components and related proteins, the post-translational mechanisms of inflammasome activation, and advances in the understanding of the structural basis of inflammasome activation.

## Introduction

Inflammasomes are signaling platforms crucial to the innate immune response to infectious diseases. These large, multimeric complexes form in response to molecular patterns unique to pathogens and cellular damage, triggering a cascade of downstream responses, including the induction of pyroptotic cell death and release of proinflammatory cytokines.^1^ Some inflammasomes directly recognize these patterns, while others indirectly sense these patterns through changes in the homeostatic environment of the cell. At the most simplistic level, an inflammasome is comprised of a sensor, an adapter, and an effector. Typically, inflammasome complexes are named after their sensor. The most well-established inflammasomes include the nucleotide-binding oligomerization domain-like receptor (NLR)-family, pyrin domain (PYD)-containing 1 (NLRP1); NLR-family, PYD-containing 3 (NLRP3); NLR-family apoptosis inhibitory protein (NAIP); NLR-family, caspase activation and recruitment domain (CARD)-containing 4 (NLRC4); absent in melanoma 2 (AIM2); and Pyrin inflammasomes. In addition, a number of other NLR-family proteins, including NLRP2, NLRP6, NLRP7, NLRP9, and NLRP12, among others, have been proposed to serve as sensors in inflammasome complexes.^2–7^ Several of these sensors use the apoptosis-associated speck-like protein containing a CARD (ASC) as an adapter molecule. The ASC adapter serves as a bridge, connecting the sensor to the downstream effector, caspase-1 (CASP1). After inflammasome assembly, CASP1 becomes activated through proximity-induced autoproteolysis. Active CASP1 is then free to process the immature forms of proinflammatory cytokines interleukin-1β (IL-1β) and IL-18 into their mature signaling forms.^8,9^ Additionally, CASP1 cleaves the protein gasdermin D (GSDMD).^10^ After cleavage, the N-terminal fragment of GSDMD (N-term GSDMD) oligomerizes to form pores in the cell membrane, allowing IL-1β and IL-18 to leave the cell and effectively executing pyroptotic, or inflammatory, cell death.^10–13^

Though these complexes serve vital functions in host defense, aberrant activation of inflammasomes has been linked with multiple diseases. Mutations within NLRP3 and Pyrin lead to the development of autoinflammatory diseases, including cryopyrin-associated periodic syndromes (CAPS) and familial Mediterranean fever (FMF).^14–18^ Additionally, chronic inflammasome activation has been tied to the development of metabolic syndromes, neurodegenerative diseases, and cancer progression.^19–21^ It is therefore unsurprising that inflammasome activation is a tightly regulated process. A number of transcription factors have been found to regulate the expression of inflammasome components, downstream effector molecules, and the upstream regulatory molecules required for successful activation. Furthermore, activation of each inflammasome is regulated by mechanisms unique to the sensing molecule. Broadly, these activation mechanisms can be divided into those reliant on direct ligand-binding and indirect mechanisms of activation. Recent structural studies have provided mechanistic details into the activation of different inflammasomes. Here, we break down the regulation of well-established inflammasomes into three separate areas to be discussed: the transcriptional regulation of inflammasome components, upstream regulators, and downstream effectors; the post-translational mechanisms of inflammasome activation; and the structural basis of inflammasome activation.

## Transcriptional regulation of inflammasomes

**Fig. 1:**
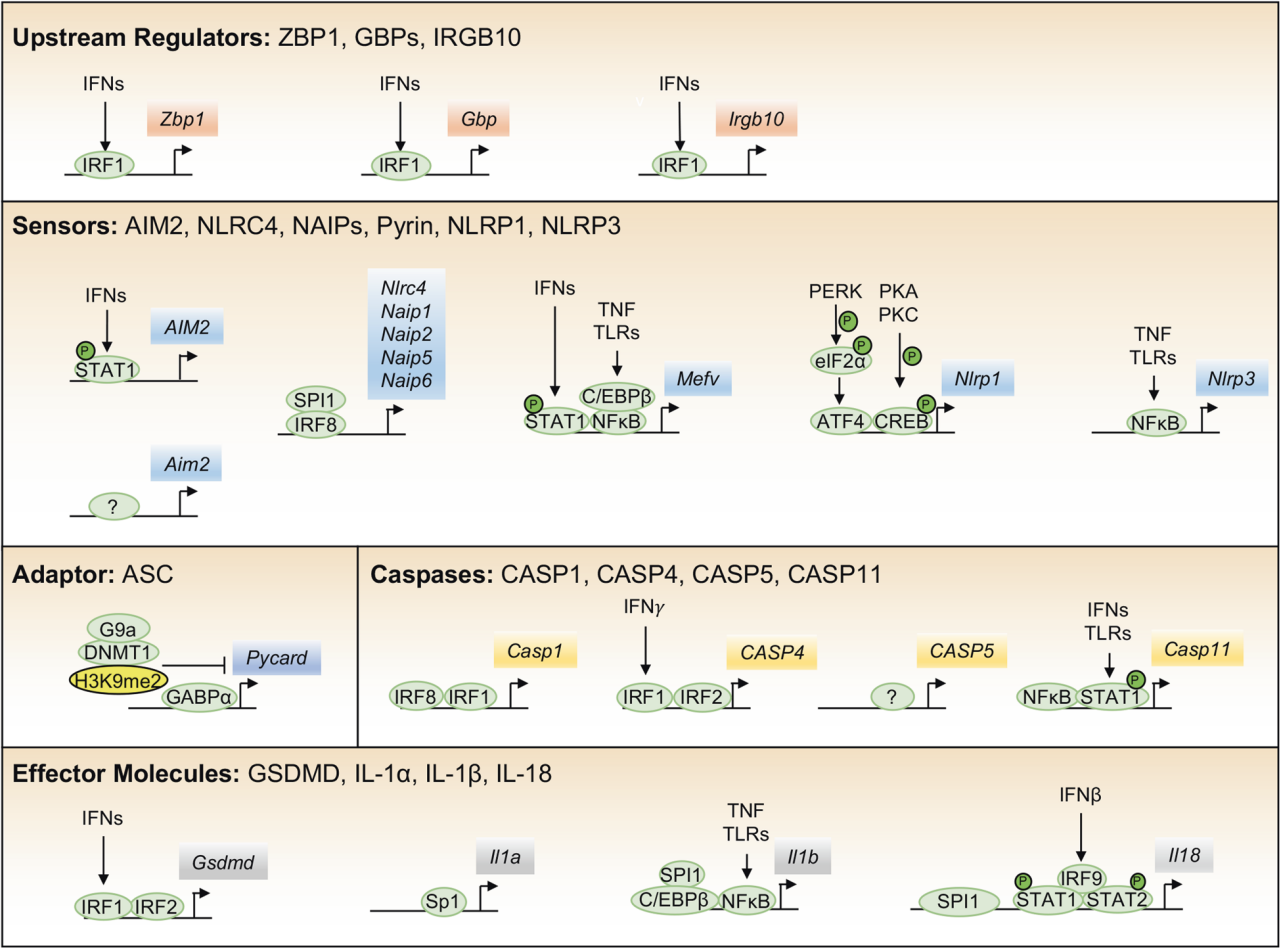
Proposed transcriptional regulators of inflammasome components. Inflammasome activity is tightly regulated at the transcriptional level, and a number of factors have been proposed to regulate the expression of inflammasome components, molecules required upstream of activation, and downstream effector molecules.

Numerous proteins have been reported to be involved in the regulation of the inflammasome response to damage and infection. Here, we discuss the transcriptional regulation of several proteins that are required upstream of inflammasome activation, directly participate in the inflammasome complex, or are crucial downstream effector molecules (Fig. 1).

### Upstream regulatory molecules

Interferon regulatory factor 1 (IRF1) has emerged as a key factor regulating the transcription of a number of molecules upstream of the activation of several inflammasomes. One such molecule is the interferon (IFN)-inducible Z-DNA-binding protein 1 (ZBP1), also referred to as DNA-dependent activator of IFN-regulatory factors or DAI, which senses intracellular viruses and assembles a unique complex that mediates parallel necroptosis, apoptosis, and NLRP3-mediated pyroptosis pathways.^22^ During influenza infection, *Zbp1* is primarily upregulated by IRF1 following IFN production.^23^ Partial expression of *Zbp1*, even in the absence of IRF1, suggests other factors may also play a role downstream of IFN signaling. During intracellular bacterial infection, IRF1 is required to upregulate a set of IFN-stimulated genes, including *Gbp2*, *Gbp5*, and *Irgb10*. These guanylate-binding proteins (GBPs) and immunity-related GTPase family member b10 (IRGB10) must be expressed to lyse the bacterial membrane or increase the exposure of transfected cytosolic lipopolysaccharide (LPS) or bacterial outer membrane vesicles for sensing by caspase-11 (CASP11) and downstream activation of the NLRP3 inflammasome.^24–28^ Additionally, these proteins are required to lyse intracellular bacteria such as *Francisella novicida* to free the bacterial dsDNA substrate for sensing by the AIM2 inflammasome.^25,26^ Immune recognition of the fungal pathogen *Aspergillus fumigatus* via activation of C-type lectin receptors (CTRs), Syk, and the nuclear factor kappa B (NF‐κB) pathway leads to increased expression of *Irf1*. Concomitant recognition via Toll-like receptors (TLRs) is required for the TLR adapter MyD88 to associate with IRF1, mediating its translocation to the nucleus and the subsequent expression of *Irgb10*. Without both the CTR and TLR activation, IRGB10 is poorly expressed, and NLRP3 inflammasome activation is reduced during *A. fumigatus* infection.^29^

### Components of the inflammasome complex

#### AIM2

In mice, AIM2 is constitutively expressed in macrophages, while in humans, it is upregulated by IFNs and signal transducer and activator of transcription 1 (STAT1) binding to a unique endogenous retroviral insertion sequence −220 bp upstream of *AIM2.*^30^ This variance in regulation likely plays an important role in differences between humans and mice that requires further investigation. As mentioned previously, transcriptional control of additional key upstream regulators of the AIM2 inflammasome further governs activation. For activation of the AIM2 inflammasome to occur during infection with intracellular bacteria such as *F. novicida*, IFNs must also upregulate IRF1 to induce GBPs and IRGB10 to carry out their roles in liberating bacterial ligands to be sensed by the inflammasomes.^24–26^ Together, multiple layers of IFN-mediated signaling tightly regulate activation of the AIM2 inflammasome.

#### NAIPs and NLRC4

Until recently, no transcription factors were known to regulate the expression of the NAIP or NLRC4 proteins necessary for NLRC4 inflammasome activation. The transcription factor IRF8 (IFN regulatory factor 8), essential for development of multiple myeloid lineage cell types, has been found to be required for the basal expression of several murine NAIP proteins and murine NLRC4. *Naip2* and *Naip5* contain IRF8 consensus sequences at −590 and −390 bp upstream of their start sites, respectively, while *Nlrc4* contains an IRF8-binding site within an intronic sequence.^31^ Data from an earlier chromatin immunoprecipitation sequencing (ChIP-seq) study also found an enrichment of IRF8-binding sites in the promoter regions of *Naip2, Naip5*, and *Naip6*, further supporting these findings.^32^ In addition to the IRF8 sites, SPI-1-binding sites are enriched in *Naip1, Naip2, Naip5*, and *Naip6*, suggesting SPI-1 and IRF8 may cooperate to regulate expression of key components of the NLRC4 inflammasome.^33^

#### Pyrin

The gene encoding the protein Pyrin, *Mefv*, is upregulated after LPS, tumor necrosis factor (TNF), or IFN treatment.^34,35^ Following stimulation with IFNγ, STAT1 binds to the gamma-activated sequence (GAS) element in the *Mefv* promoter at −731 bp and rapidly upregulates *Mefv.*^34^ TNF signaling was shown to promote human *MEFV* expression in a CCAAT enhancer-binding protein β (C/EBPβ) and NF‐κB p65-dependent manner through binding of C/EBPβ and NF‐κB to conserved sites at −163 and −55 bp. In a mouse model of FMF, TNF signaling through TNF receptor 1 (TNFR1) was critical for driving pathogenic overexpression of *Mefv*, supporting earlier studies.^36,37^ Other putative transcription factor-binding sites have been identified but are poorly studied, including those for SPI-1, AP-1 (c-Jun/c-Fos), and Runt family transcription factors.^34^ Patients with mutations associated with FMF display varying severity of disease, and mutations in the promoter region (c.−614C>G or c.−382C>T) are associated with decreased or increased *MEFV* expression, respectively, suggesting that differential gene expression may play a clinically important role in FMF.^38^

#### NLRP1

Humans possess a single NLRP1, whereas mice have three paralogues, NLRP1a, b, and c, with NLRP1c being a pseudogene. In human cells, endoplasmic reticulum (ER) stress activates inositol-requiring enzyme 1 α (IRE1α) and protein kinase R-like ER kinase (PERK), which upregulates activating transcription factor 4 (ATF4), allowing it to bind the *NLRP1* promoter and increase *NLRP1* expression.^39^ Another study showed that protein kinase A (PKA)/PKC and cAMP response element-binding protein (CREB) also regulated NLRP1 expression in myeloid leukemia cells; however, in HeLa cells this CREB-dependent NLRP1 expression was not observed, suggesting there may be cell type-specific regulation.^39,40^ In neurons, NLRP1 was repressed by heme oxygenase-1 (HO-1)-dependent inhibition of ATF4 expression, and overexpression of HO-1 limited neuronal death and damage in a spinal cord injury model, supporting the proposed role for ATF4 in directly regulating *NLRP1* expression.^41^

#### NLRP3

Numerous inflammatory diseases are driven by activation of the NLRP3 inflammasome, and a large number of upstream triggers are implicated in the transcriptional priming of NLRP3. Early studies identified a critical role for NF‐κB in the upregulation of *Nlrp3* following inflammatory signaling through the TLRs and their adapter proteins (MyD88, Toll-interleukin receptor domain-containing adapter protein inducing IFNβ (TRIF)) and downstream kinases (IRAK1/4).^42,43^ The NF‐κB-binding sites (−1330 to −1292 bp and −1238 to −1228 bp) in the *Nlrp3* promoter were experimentally determined by electrophoretic mobility shift assay (EMSA) and ChIP assays.^44^ Additionally, signaling downstream of the TNF receptor, Fas-associated protein with death domain (FADD), and caspase-8 (CASP8) can also positively regulate NF‐κB-dependent *Nlrp3* transcription.^42,43,45,46^ A further study found that genetic loss of TNF in mice led to a downregulation of NLRP3 mRNA and the mRNA of other inflammasome components. Additionally, loss of TNF helped to protect against disease induced by NLRP3 mutations.^47^ While NF‐κB-dependent transcriptional regulation of *Nlrp3* is the most well studied, other putative transcription factor-binding sites have been identified in the promoter region including Sp1, c-Myb, AP-1, and c-Ets, and disruption of the Sp1 site leads to a reduction in *NLRP3* expression.^48^ An additional study in human endothelial cells also found that NLRP3 was regulated by the sterol regulatory element-binding transcription factor 2 (SREBP2) binding between −1379 and −1368 bp.^49^ Negative regulation of *Nlrp3* has also been observed following interaction of growth factor independence 1 (GFI1) with the promoter Gli-response element 1 (GRE1), which repressed expression of *Nlrp3* and transcriptional activity of NF‐κB p65.^50^ Post-transcriptionally, the expression of NLRP3 has been shown to be regulated by microRNAs.^51,52^ Given the diversity of activating signals for NLRP3, tight transcriptional regulation is likely an important checkpoint to preserve cell viability.

#### ASC

Multiple inflammasome sensors containing a PYD require the adapter protein ASC (encoded by *Pycard*) to recruit and activate CASP1. ASC is constitutively expressed in many cells and does not appear to be inducible by inflammatory signaling. Earlier studies identified ASC both through its ability to form protein aggregates during HL60 cell death, leading to the name ASC, and as a gene that is highly methylated in human breast cancers, leading to the name target of methylation-induced silencing (TMS1).^53–55^ Methylation-induced silencing of ASC was found to be important in many other cancer cell lines, and a recent study found that a long noncoding antisense RNA called PYCARD-AS1 localized to the *PYCARD* promoter, facilitating DNA methylation and H3K9me2 modification via DNA methyltransferase 1 (DNMT1) and G9a recruitment.^56–59^ The ASC promoter is found within a large CpG island that is regulated by histone modifications. Basal expression of ASC appears to be regulated by the Ets family member GA-binding protein α (GABPα), which is enriched at a DNase I hypersensitive site (HS2) intronic element in the ASC promoter.^60^ Given the important role that ASC plays in inflammasome activation and, in some cases, apoptotic cell death, a better understanding of its regulation may provide insight into how cancers and autoinflammatory diseases progress.

### Caspases

#### CASP1

The transcriptional regulation of CASP1 is poorly understood. In murine macrophages, CASP1 is constitutively expressed, with some reports suggesting that CASP1 may be upregulated by NF‐κB or IRF1. Indeed, the *Casp1* promoter contains putative NF‐κB- and IRF1-binding sites, and CASP1 is upregulated in chronic inflammatory conditions.^61^ Another study showed that IRF8 binding to an IRF8 consensus binding site at −40 to −31 bp upstream of the *CASP1* start site regulated promoter activity in B cells, and that IRF1 synergistically acts with IRF8 to regulate *CASP1* expression.^62^ Less well studied factors that regulate CASP1 include Ets1, p63, and p73.^63–65^ Given the critical role CASP1 plays in pyroptosis, it is important to understand how it is regulated at the basal level and in inflammatory diseases.

#### CASP4/5/11

Intracellular sensing of LPS is mediated by either caspases-4/5 in humans (CASP4 and CASP5, respectively) or CASP11 in mice. The expression of *Casp11* can be induced by TLR4/TRIF-mediated activation of NF‐κB or by IFN-mediated activation of STAT1. These molecules bind to putative sites at −119 bp and −17 to −59 bp in *Casp11*, respectively.^66–70^ The NF‐κB-mediated regulation of *Casp11* expression after LPS stimulation was inhibited when poly(ADP-ribose) polymerase-1 (PARP1) was knocked down, suggesting that it acts as a transcriptional cofactor for NF‐κB.^71^ Another proposed transcriptional regulator of *Casp11* is the C/EBP homologous protein (CHOP), which is important for upregulation following LPS-induced lung injury in mice.^72^

Human CASP4 and CASP5 both mediate pyroptosis downstream of recognition of intracellular LPS, but the relative contribution of each is still poorly understood. CASP4 and CASP5 proteins are constitutively expressed in monocytes, unlike the low basal expression of CASP11 in murine macrophages.^73^ In human HT-29 colon carcinoma cells, IFNγ upregulated mRNA levels of *CASP1* and *CASP5*, but not *CASP4*; however, CASP5 protein levels were not increased. In human monocyte THP-1 cells, CASP5 mRNA and protein levels were increased by LPS stimulation.^74^ Another study found that *CASP5* was expressed in psoriatic skin and was regulated by IFN and NF‐κB.^75^ In primary human monocytes, the mRNA of *CASP4* and *CASP5* was transiently increased by LPS, but the protein levels were not changed, suggesting that there are important cell type-specific mechanisms that regulate these proteins.^73^ More recently, a critical regulator of *CASP4*, IRF2 (IFN regulatory factor 2), was identified by CRISPR/Cas9 screening of human U937 monocyte cells and iPSC-derived monocytes.^76^ The loss of expression of *CASP4* in IRF2 knockout cells could be rescued by IRF1-mediated transcription following priming with IFNγ.^76^ In a similar screening study, IRF2 and IRF1 both appeared to be dispensable for *CASP4* expression in a human endothelial cell line EA.hy926, further suggesting that there is cell type-specific regulation of these LPS-responsive caspases.^77^

### Downstream effector molecules

#### GSDMD

The executioner molecule of pyroptosis, GSDMD, is constitutively expressed and autoinhibited in myeloid cells until activation by caspase cleavage. Recently, the transcription factor IRF2 was identified as a key regulator of basal *Gsdmd* expression in murine macrophages. Similar to the IRF2-dependent regulation of human *CASP4*, IFNγ priming of IRF2-deficient cells could rescue *Gsdmd* expression via IRF1.^76,77^ Basal GSDMD expression in U937 cells did not appear to require IRF1 or IRF2, in contrast with what was observed in the human endothelial cell line EA.hy926, but deletion of both IRF1/2 in U937 cells rendered them insensitive to IFNγ-mediated upregulation of *GSDMD.*^76^ It is still unclear whether variation in the regulation of IRF2 and GSDMD, which has been identified between individuals, corresponds to differences in immune responses to Gram-negative bacteria and septic shock.^78^ Together, these studies establish a critical role for IRF1 and IRF2 in regulation of human GSDMD.

#### IL-1α, IL-1β, IL-18

Inflammasome activation ultimately leads to lytic cell death and, in most cases, cleavage-induced maturation of the cytokines IL-1β and IL-18. Unlike IL-1β, IL-1α does not need to be cleaved to functionally signal through the IL-1 receptor (IL-1R). The human *IL1A* promoter contains an Sp1-binding site at −52 to −45 bp and an AP-1 site at −13 to −5 bp as well as NF‐κB-binding sites that mediate expression in many cell types.^79–82^ An antisense transcript of IL-1α, AS-IL-1α, also positively regulates IL-1α expression by facilitating recruitment of RNA polymerase II to the *Il1a* promoter.^83^ Rapamycin has been shown to negatively regulate the expression of IL-1α, disrupting inflammation associated with senescence.^84^ Associations between IL-1α single nucleotide polymorphisms (SNPs) and disease have been documented and are likely to influence the basal expression of this important inflammatory cytokine.

In contrast to IL-1α, which is expressed in many different cell types, IL-1β is expressed in myeloid cells in a highly inducible manner downstream of TLRs.^85^ The promoter of *IL1B* contains a classical TATA box (−31 bp), 2 putative CAAT boxes (−125 and −75 bp), and binding sites for Sp1, SPI-1, C/EBPβ, CREB, and NF‐κB.^86–91^ Similar to IL-1α, SNPs associated with disease progression have been identified in regulatory regions of IL-1β (National Center for Biotechnology Information), requiring future study into their contributions to diseases.

In addition to IL-1β and GSDMD, the cytokine IL-18 requires cleavage by CASP1 to become functional. IL-18 is constitutively expressed in murine bone marrow-derived macrophages (BMDMs) and human peripheral blood mononuclear cells (PBMCs), but can also be upregulated following IFN priming through STAT1/2 and IRF9.^92–94^ SNPs in the *IL18* promoter are associated with inflammatory diseases, suggesting they lead to changes in gene transcriptional regulation.^95,96^ The expression of *Il18* in mice requires SPI-1 binding at −31 to −13 bp and IRF8 binding at −39 to −22 bp, and IRF1 associates with IRF8 following IFNγ stimulation.^97,98^ An AP-1 site was also identified at −1120 to −1083 bp that promotes IFNγ-induced and basal expression of IL-18.^85^ Another study found that human IL-18 could be regulated by STAT5 binding at multiple promoter locations and that a potential Bcl6 repressor-binding site may compete for binding by STAT5.^99,100^ Additionaly, human IL-18 does not possess an IRF8 consensus binding site, suggesting it may be regulated differently than murine IL-18.^101^

## Post-translational mechanisms of activation

Each inflammasome recognizes and becomes activated in response to unique signals. This can occur through a direct interaction between a ligand and the sensor of an inflammasome or indirectly through sensing of the cellular environment by an inflammasome. In addition, a number of post-translational modifications regulate the activation of the different inflammasome sensors. These post-translational modifications are summarized in Table 1.

**Table 1:** Post-translational modifications regulating inflammasome activity.

Inflammasome	Modification	Site	Enzyme or trigger	Effect on activity	References
NLRP1	Cleavage	S1213	NLRP1	Positive	^112^
Pyrin	Phosphorylation	S242	PKN1/2	Negative	^119,120^
NLRP3	Phosphorylation	S5 (h) S3 (m)	PP2A	Negative	^175^
		S198 (h) S194 (m)	JNK	Positive	^176^
		S295 (h) S293 (m)	PKD	Positive	^177^
		S295 (h) S291 (m)	PKA	Negative	^178,179^
		Y861 (h) Y859 (m)	PTPN22	Positive	^180^
	Ubiquitination	LRR domain	BRCC36 (h) BRCC3 (m)	Negative	^181^
		K689 (h)	FBXL2	Negative	^182^
		PYD	TRIM31	Negative	^183^
		NBD/LRR domain	MARCH7	Negative	^184^
		NBD	ARIH2	Negative	^185^
	Alkylation	C419 (h)	Acrylamide derivatives	Negative	^186^
	S-nitrosylation	Unknown	NO and SNAP	Negative	^187^

*ARIH2* Ariadne RBR E3 ubiquitin protein ligase 2, *BRCC3* lysine-63–specific deubiquitinase, *BRCC36* lysine-63–specific deubiquitinase, *FBXL2* F-box and leucine rich repeat protein 2, *h* human, *JNK* c-Jun N-terminal kinase, *LRR* leucine rich repeat, *m* mouse, *MARCH7* membrane associated ring-CH-type finger 7, *NBD* nucleotide binding domain, *NO* nitric oxide, *PKA* protein kinase A, *PKD* protein kinase D, *PP2A* protein phosphatase 2A, *PTPN22* protein tyrosine phosphatase non-receptor type 22, *PYD* pyrin domain, *SNAP* S-nitroso-N-acetylpenicillamine, *TRIM31* tripartite motif containing 31.

### Activation of AIM2 and NAIP–NLRC4 by ligand binding

**Fig. 2:**
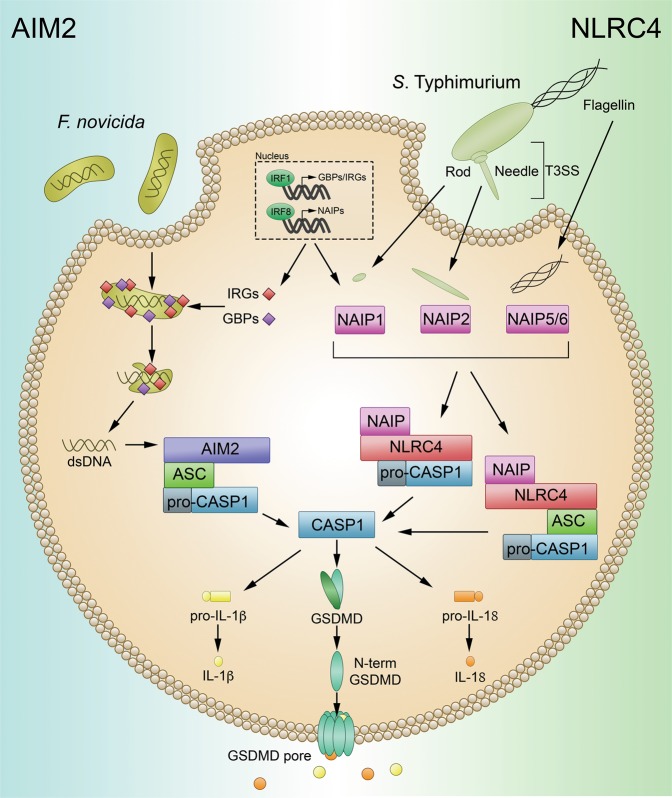
Activation of AIM2 and NAIP–NLRC4 by direct ligand binding. ***AIM*2:** GBPs and IRGs mediate the bacteriolysis of intracellular bacteria, including *F. novicida*, leading to the exposure of dsDNA. Binding of dsDNA to AIM2 leads to AIM2 inflammasome activation. Active AIM2 recruits and activates CASP1 in an ASC-dependent manner to initiate pyroptosis through GSDMD and proinflammatory cytokine cleavage. ***NAIP–NLRC4:*** T3SS and flagellin proteins activate different murine NAIPs through direct binding interactions. NAIPs then activate NLRC4, which directly interacts with CASP1, leading to its autoactivation and the induction of pyroptosis.

The NAIP–NLRC4 and AIM2 inflammasomes are activated directly in response to the binding of a pathogen-associated ligand. Activation of the AIM2 and NAIP–NLRC4 inflammasomes is summarized in Fig. 2.

#### AIM2

The AIM2 inflammasome initiates pyroptosis in response to double-stranded DNA (dsDNA). The hematopoietic expression, IFN inducibility, nuclear localization (HIN)-200 domain of the AIM2 protein directly binds dsDNA within the cytosol, leading to the association of AIM2 with ASC and subsequent activation of the AIM2 inflammasome.^102^ Activation of AIM2 in response to intracellular bacteria requires IFN-inducible GTPases, including GBPs and IRGB10.^26^ These proteins target intracellular bacteria, leading to bacteriolysis and the freeing of dsDNA into the cytosol for sensing by AIM2.

#### NAIP–NLRC4

NAIPs and NLRC4 work cooperatively to mediate inflammasome activation.^103–107^ NAIPs are responsible for directly sensing bacterial ligands.^103–105^ Humans have a single NAIP capable of sensing the needle protein of the bacterial type 3 secretion system (T3SS) and flagellin proteins, while mice have several NAIPs. Like human NAIP, murine NAIP1 recognizes the needle protein of the T3SS, while murine NAIP2 recognizes the T3SS rod protein. NAIP5 and NAIP6 from mice recognize and bind bacterial flagellin proteins.^103,104,107^ The ligands of the remaining murine NAIPs remain unknown. The ligand-bound NAIP complex acts as a nucleation point by recruiting and activating NLRC4, which in turn activates CASP1. The CARD of NLRC4 allows direct interaction between NLRC4 and CASP1. However, NLRC4 is still capable of interacting with ASC and has been shown to localize to the ASC speck.^108^ In addition, some reports have found that flagellin leads to the phosphorylation of NLRC4 at Ser533, which primes NLRC4 for activation.^109,110^ In contrast, a recent study suggests that phosphorylation of Ser533 plays no role in the activation of NLRC4.^111^

### Indirect activation of NLRP1 and Pyrin

**Fig. 3:**
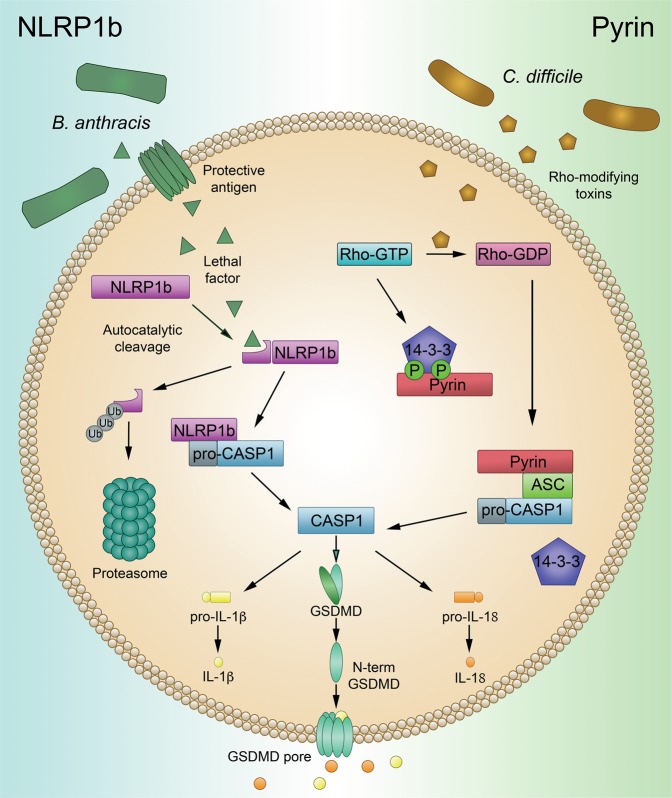
Activation of NLRP1b and Pyrin through indirect mechanisms. ***NLRP1b:*** NLRP1b undergoes auto-processing at the FIIND domain to form two peptide fragments that continue to interact. NLRP1b-activating stimuli, such as LF from *B. anthracis*, cleave the N-terminal fragment of NLRP1b, targeting this fragment for ubiquitination and subsequent degradation. Degradation of the N-terminal fragment of NLRP1b relieves the C-terminal fragment from autoinhibition and allows the NLRP1b inflammasome to activate. ***Pyrin:*** Under homeostatic conditions, Pyrin is kept inactivated through phosphorylation-mediated interactions with 14-3-3. Rho-modifying toxins, such as TcdB from *C. difficile*, lead to the inactivation of Rho-GTPase. Loss of Rho-GTPase activity leads to unphosphorylated Pyrin. This allows Pyrin to disassociate from 14-3-3 and become active.

In contrast to the AIM2 and NAIP-NLRC4 inflammasomes, the conformational changes that induce NLRP1 and Pyrin activation do not occur as the result of direct ligand binding. The NLRP1 inflammasome is regulated through a unique process of functional degradation, while the Pyrin inflammasome senses the inactivation of other host proteins (Fig. 3).

#### NLRP1

NLRP1 was the first pattern recognition receptor that was found to be capable of forming an inflammasome complex.^1^ Encoded by a single *NLRP1* gene in humans and three (*Nlrp1a-c*) genes in mice, both human NLRP1 and the mouse paralogues possess a unique function to find (FIIND) domain. Self-cleavage at this FIIND domain in NLRP1 is required for later inflammasome function.^112^ Under normal cellular conditions, the two fragments of NLRP1 continue to interact, resulting in the sensor remaining autoinhibited. A subsequent cleavage event of the N-terminus by a pathogenic trigger, such as cleavage of murine NLRP1b by lethal factor (LF) from *Bacillus anthracis*, results in ubiquitination and degradation of the now unstable fragment by the E3 ubiquitin ligase UBR2.^113–115^ This functional degradation event relieves autoinhibition of the C-terminal fragment of the protein, allowing it to oligomerize and form an activated inflammasome complex.^113–115^ In addition, inhibition of host serine proteases DPP8/9 leads to a similar proteasomal-dependent activation of multiple variants of NLRP1, including those not sensitive to anthrax LF.^116–118^

#### Pyrin

Activation of the Pyrin inflammasome is also controlled without direct binding between Pyrin and a bacterial ligand. Under normal conditions, Pyrin is phosphorylated by protein kinase 1 (PKN1) and 2 (PKN2), and this phosphorylation event promotes the association of Pyrin with the 14-3-3 proteins, blocking Pyrin activation.^119^ Bacterial toxins, including the TcdB toxin from *Clostridium difficile*, inactivate host Rho-GTPase.^120^ Subsequently, PKN1 and PKN2 become inactivated, resulting in the loss of Pyrin interaction with 14-3-3 proteins and inflammasome activation.^119,120^ Thus, the Pyrin inflammasome senses pathogens through the modifications of other host proteins.

### Activation of NLRP3

Currently, there is no known unifying mechanism of activation for the NLRP3 inflammasome. It therefore remains unclear if NLRP3 is activated by a specific ligand or through indirect means. Numerous triggers have been reported to activate the NLRP3 inflammasome, including ATP, potassium efflux, lysosomal disruption by crystalline particulates, mitochondrial DNA, and the generation of reactive oxygen species.^121–125^ Due to the sheer number of stimuli capable of activating NLRP3, and the differences in their structural and chemical properties, it is unlikely that a single ligand exists for this inflammasome. Instead, NLRP3 activation is likely regulated in an indirect manner reflecting the homeostatic nature of the cell. This notion is supported by a recent study establishing a novel regulator of NLRP3 activation, DDX3X, a protein normally involved in stress granule formation.^126^ By exploring the crosstalk between cellular stress and pyroptotic cell death, the authors found that DDX3X was required for NLRP3 activation and that sequestration of DDX3X by the formation of stress granules inhibited this inflammasome’s activation.^126^ Thus, DDX3X represents a cellular life or death checkpoint, whereby the fate of the cell is decided by DDX3X’s recruitment to either the stress granule or inflammasome complex. NLRP3’s role as a homeostatic sensor is further supported by the apparent requirement for NEK7, a kinase normally involved in mitosis, in activation.^127–129^

Numerous models have been proposed to describe the common, upstream event that activates NLRP3 in response to such diverse stimuli. Potassium ion efflux has been shown to activate NLRP3 in response to an number of triggers and has been proposed to be the common mechanism of NLRP3 activation.^130,131^ Different activators, including the formation of membrane pores by toxins, facilitate potassium efflux capable of initiating NLRP3 activation.^130,131^ However, activation of NLRP3 through small molecules targeting mitochondria was found to be potassium efflux-independent.^132^ In addition, reactive oxygen species and oxidized mitochondrial DNA have been shown to activate the NLRP3 inflammasome, highlighting the importance of the mitochondria in NLRP3 activation.^123–125,133,134^ Recently, disruption of the trans-Golgi network (TGN) has been described as a cellular event capable of activating NLRP3 in response to potassium-dependent and potassium-independent NLRP3 activators.^135^ The authors found that a polybasic stretch of four lysine residues on NLRP3 mediated charge-based interactions with phosphatidylinositol-4-phosphate, a component of the TGN. Interactions between these molecules led to the clustering of NLRP3 and subsequent assembly of ASC filaments at the disrupted TGN.^135^ These findings provide new avenues of exploration for the field as the mechanisms of the common upstream event responsible for NLRP3 activation continue to be sought.

In many circumstances, normal, basal levels of the NLRP3 protein within the cell are insufficient to trigger strong inflammasome activation. Thus, the current model of canonical NLRP3 activation requires a two-step process. Signal 1, known as the priming signal, leads to the transcriptional upregulation of NLRP3 inflammasome components and related molecules described earlier in this review, as well as post-translational modifications of NLRP3 that regulate activation (Table 1). After priming, a subsequent activating signal is required. At this point, NLRP3 oligomerizes and binds ASC, and inflammasome activation proceeds. FADD and CASP8 have been shown to play crucial, multifaceted roles in this two-step model.^45^

**Fig. 4:**
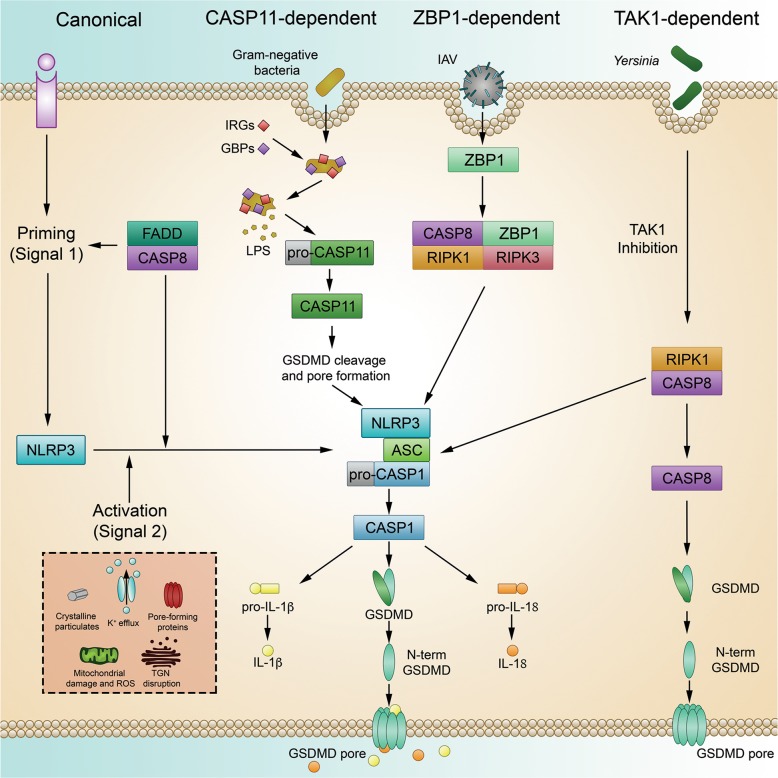
Activation of the NLRP3 inflammasome. In response to a wide range of stimuli and live pathogens, NLRP3 becomes activated through canonical, CASP11-dependent, ZBP1-dependent, and TAK1-dependent mechanisms.

In addition to the large number of reported NLRP3 stimuli, there is an expansive cohort of live pathogens that activate the NLRP3 inflammasome through a number of distinct upstream pathways (Fig. 4).^22,136–140^ In the classical, or canonical pathway, activation of NLRP3 proceeds in the two-step manner. *A. fumigatus* appears to activate NLRP3 in murine macrophages through this canonical mechanism by initially engaging innate immune receptors to prime the cell, then activating the inflammasome after IRGB10-depedent ligand release.^29^

Activation of NLRP3 by Gram-negative bacteria or cytosolic LPS requires a series of cellular events that are unique from canonical activation. Referred to as non-canonical, this mechanism of NLRP3 inflammasome activation is dependent on CASP11 in mice and CASP4 and CASP5 in humans.^73,136^ After activation by LPS, CASP4/5/11 proceed to cleave GSDMD, triggering pyroptosis.^73,141,142^ The pores formed by CASP11-activated GSDMD lead to ionic flux in the cell, including potassium efflux, which induces NLRP3 activation, propagating the inflammatory signal.^143^ Similar to their role in liberating dsDNA for sensing by AIM2, IFN-regulated GBPs and IRGB10 are required in mice to effectively free LPS from vacuole-resident or cytosolic intracellular Gram-negative bacteria for recognition by CASP11.^26^

In the context of influenza infection, the innate immune sensor ZBP1 is emerging as a key regulator of the concurrent induction of NLRP3-dependent pyroptosis, mixed-lineage kinase domain-like pseudokinase (MLKL)-dependent necroptosis, and CASP8-dependent apoptosis. Through RIP homotypic interaction motif (RHIM) domains, ZBP1 interacts with RIPK3 (receptor-interacting serine/threonine-protein kinase 3) and forms a multi-component complex that regulates the induction of these parallel cell death pathways.^144^ Though the specific ZBP1-activating ligands are currently unknown, recent studies have demonstrated that IFN-induced ZBP1 is required for influenza-induced NLRP3 activation.^22,145,146^

Transforming growth factor β-activated kinase 1 (TAK1) has been shown to control NLRP3 activation that appears to be independent of the canonical two-step activation, non-canonical CASP11-mediated activation, and IAV-induced ZBP1-mediated activation. Loss of TAK1 leads to the spontaneous activation of NLRP3 in a RIPK1-dependent manner, leading to the subsequent induction of cell death.^147^ Further studies have found that TAK1 inhibition after *Yersinia* infection leads to the CASP8-dependent cleavage of GSDMD and GSDME in a RIPK1-dependent manner.^148–150^ While there are still many unanswered questions regarding TAK1-mediated regulation of NLRP3 and cell death, these and other studies help to highlight the complexity of communication between cell death pathways. The unveiling of ZBP1 and TAK1 as master regulators of pyroptosis, apoptosis, and necroptosis, along with the rapidly expanding evidence of communication between these pathways, has led to the concept of PANoptosis, in which a putative PANoptosome acts as a central cell death complex that can initiate all three of these pathways.^151,152^ The convergence of these three programmed cell death pathways and the identification of master regulators controlling all three is intriguing and could be powerful therapeutically. Future research will be needed to determine the therapeutic potential of these master regulators and to unravel the complexities of this central PANoptotic process, including detailed exploration of the mechanisms of this multifaceted cell death.

## Structural basis of inflammasome activity

**Fig. 5:**
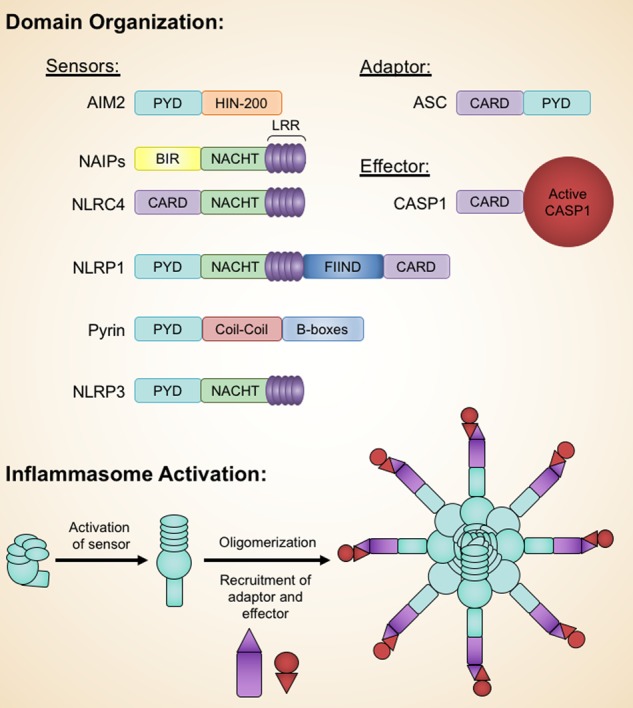
Domain organization and basic inflammasome assembly. The domains of AIM2, NAIPs, NLRC4, NLRP1, Pyrin, NLRP3, ASC, and CASP1 are shown. The archetype inflammasome structure is comprised of a sensor, adapter, and effector protein. After activation, the sensor oligomerizes and recruits the adapter and effector proteins to the inflammasome complex. LRR, leucine rich repeat; BIR, baculoviral inhibitor of apoptosis protein repeat.

At the molecular level, assembly of inflammasome signaling complexes is mediated through interactions between homotypic protein domains (Fig. 5). Several inflammasomes contain a PYD, responsible for the recruitment of the adapter protein ASC. After activation, inflammasomes utilizing this adapter will interact with ASC through PYD–PYD interactions, forming large, filamentous oligomers.^153–155^ ASC, comprised of an N-terminal CARD and C-terminal PYD, will then recruit CASP1 through CARD–CARD interactions. NLRP1 and NLRC4 are thought to directly interact with CASP1 through CARD–CARD interactions, though these NLRs could also interact with ASC through CARD–CARD interactions. Both PYD and CARD belong to the Death Domain (DD) superfamily and exhibit structural characteristics common to members of this family.^156–158^ The helical nature of members of the DD family leads to the formation of filamentous, higher-order complexes that allow for signal amplification and allosteric activation of executioner molecules, such as CASP1. In innate immunity, the propagation of signals is often mediated through the formation of these higher-order complexes, known as supramolecular organizational complexes (SMOCs).^159,160^ SMOCs utilize organellar membranes as scaffolds for formation and function by increasing the local concentration of signaling molecules to surpass a response threshold.^159,160^

Structural studies of inflammasome components by X-ray crystallography and nuclear magnetic resonance (NMR) have yielded a number of valuable insights. However, the heterogeneous, filamentous nature and size of the inflammasome complexes make analysis by these methods difficult. In recent years, advances in cryo-electron microscopy (cryo-EM) have led to a number of novel findings in the field of inflammasome activation. Such studies have revealed filamentous organization of PYDs and CARDs,^154,161^ plasticity within PYDs,^162^ and unique details of individual inflammasome activation.^129,163,164^ In addition, structural and biochemical studies have yielded mechanistic insight into the execution of pyroptotic cell death by gasdermin proteins.

Due to their more straightforward nature of activation, inflammasomes activated through direct ligand-binding mechanisms have presented attractive targets for structural researchers. As such, structural studies of the HIN-200 domain of AIM2 and the NAIP–NLRC4 inflammasome have yielded insights into the mechanistic details of activation.

### AIM2

Activation of AIM2 relies on direct binding to dsDNA mediated through interactions between the DNA and the HIN domain of AIM2. Jin et al. reported these interactions to be mediated by electrostatic interactions between the positively charged HIN domain and the negatively charged phosphate backbone of the dsDNA, based on X-ray crystallography of the HIN domain complexed with DNA.^165^ The authors also found that activation of the AIM2 inflammasome, as measured by IL-1β secretion, was dependent on the length of the dsDNA ligand used in cellular experiments, and that DNA of around 80 base pairs was required for optimal IL-1β secretion.^165^ This evidence suggests that the DNA ligand, rather than the oligomerized sensor, serves as the structural scaffold for AIM2 inflammasome activation. A further structural study of a related murine Pyrin and HIN-containing (PYHIN) family member, p202, supports this hypothesis.^166^ p202 is comprised of a HIN1 and HIN2 domain and is known to inhibit AIM2 inflammasome activation.^102^ Yin et al. found that the HIN1 domain of p202 interacts with DNA, while the HIN2 domain mediates homo-tetramerization. Additionally, the authors found that the HIN2 domain is capable of interacting with the HIN domain of AIM2. The authors suggest that this interaction occludes clustering of AIM2 on the DNA, thereby preventing recruitment of the ASC adapter protein to the AIM2 inflammasome by spatially separating the PYDs of individual AIM2 molecules.^166^

### NAIP–NLRC4

Cryo-EM studies have revealed that binding of NAIPs to their respective ligands triggers a conformational change that facilitates inflammasome activation.^163,164,167,168^ The observed rotation, occurring between helix domains within the NACHT domain, relieves autoinhibition by freeing a previously obscured NLRC4-binding region.^163,164,167,168^ Binding of an NLRC4 molecule to this newly exposed, highly charged region on the activated NAIP triggers a similar rotation in the NLRC4 NACHT domain. As with the conformational change observed in NAIPs, this rotation exposes a charged NLRC4-binding site, thus propagating self-oligomerization. Therefore, the ligand-bound NAIP serves as a nucleation point for the formation of the activated wheel-shaped NAIP–NLRC4 inflammasome, where only a single activated NAIP subunit is complexed with multiple NLRC4 subunits through NACHT interactions.

### NLRP3

The NLRP3 inflammasome has also been of high interest in the field of structural inflammasome biology for a number of years. In a recent study, Sharif et al. report a 3.8 Å cryo-EM structure of inactive NLRP3 in complex with the mitotic kinase NEK7.^129^ Here, the authors found that electrostatic interactions between a predominantly negative NLRP3 interface and a positive NEK7 interface mediate NLRP3-NEK7 interactions, and they hypothesize that NEK7 plays a role in bridging NLRP3 protomers. Biochemical analysis of site-directed mutants supports these cryo-EM findings. As the structure of NLRP3/NEK7 is in an inactivated state, the authors incorporated the cryo-EM structure of activated NLRC4 to model an activated NLRP3 complex. Utilizing this predicted model, the authors hypothesize that while NEK7 is insufficient to induce activation, it may facilitate NLRP3 oligomerization by bridging NLRP3 protomers. This first structural look at the NLRP3 inflammasome also provides valuable insight into the potential effects of CAPS-associated mutations. Future studies investigating activated NLRP3 and the effects of CAPS-related mutations on the structural assembly of the NLRP3 inflammasome will provide valuable therapeutic insight into CAPS pathogenesis.

### Gasdermin

The X-ray crystal structure of full-length GSDMA3 shows that gasdermins are kept autoinhibited through interactions between N- and C-terminal domains.^10^ Cleavage relieves this autoinhibition, freeing the cytotoxic N-terminal fragment to oligomerize and form pores of ≈18 nm in the cell membrane. Biochemical experiments and analysis of the cryo-EM structure of the activated GSDMA3 pore determined that interactions between GSDMA3 and acidic lipid head groups are necessary for pore formation.^169^ The cryo-EM density of a non-membrane-inserted pore was observed in this study, prompting the authors to propose a model of activation whereby the N-terminal fragments oligomerize before insertion into the membrane.^169^ It remains to be determined if this observed “soluble pore” is an in vitro artifact of purification or a physiologically relevant intermediate state of the gasdermin pore. In contrast, Mulvihill et al. found that insertion of the N-terminal fragment of human GSDMD into lipid membranes precedes oligomerization and pore formation.^170^ Further dissection of the mechanistic order of events in gasdermin membrane pore formation will profoundly impact our understanding of the functionality of gasdermin family proteins.

## Concluding remarks

The regulation of inflammasomes is a delicately complex process, mediated by a wide number of transcription factors, regulatory molecules, and cellular pathways. Activation of inflammasomes plays a critical role in the defense of a host against infection and sterile insults, but dysregulation of any of these regulatory processes can lead to the development and progression of disease. As such, the efficacy of therapies targeting the upstream activation of inflammasomes and the downstream consequences of inflammasome activation is being avidly explored in a number of diseases. A recent study in mice found that inducing the activation of NLRP3 in conjunction with immune checkpoint inhibitor therapy is beneficial against tumors.^171^ Conversely, a phase 3 clinical study of the drug canakinumab found that blocking IL-1β can reduce the incidences of cardiovascular disease and lung cancer.^172,173^ In addition to these studies, a number of other clinical trials exploring the role of IL-1 signaling and inflammasome activation are currently in progress or recruiting. The different outcomes of these studies highlight the context-specific roles of inflammasome activation and IL-1 signaling in different diseases, indicating that thorough future studies are required to unlock the therapeutic potential of inflammasome modulation. From a therapeutic standpoint, the unique characteristics of each inflammasome’s mechanism of activation are promising. The ability to selectively target an aberrant inflammasome, for example the Pyrin inflammasome in patients with FMF or the NLRP3 inflammasome in patients with CAPS, while leaving the others free to respond to infections and other insults, presents an attractive alternative to a total blockade of IL-1β signaling or inhibiting a shared downstream effector molecule. As such, it is crucial to develop a thorough mechanistic understanding of each of the inflammasomes.

While our understanding of the mechanisms regulating inflammasome activation has expanded tremendously, many crucial questions remain unanswered. Though it is one of the most well-studied inflammasomes, there is not a universally agreed upon model of NLRP3 activation. In addition, it is unclear what innate immune sensors, in addition to the ones discussed in this review, form true inflammasome complexes. Several reports suggest that multiple sensors can be recruited to the same complex.^108,174^ While the functional significance of heterogeneous inflammasome complexes is unclear, the concept is intriguing and will likely produce interesting studies in the future.

Further structural studies of inflammasome biology will also provide valuable insight into the conserved and unique mechanisms of inflammasome activation. It is currently unclear how the oligomerized sensors and filamentous adapter and effector regions of inflammasomes spatially interact with one another, as current structures do not include both of these regions. Future studies will likely focus on resolving these interactions. Furthermore, currently available structures suggest that in addition to having unique mechanisms of activation, different inflammasomes also have unique mechanisms of assembly. Developing a mechanistic understanding of the activation and assembly of each inflammasome at the transcriptional, post-translational, and structural levels will help guide the development of therapeutics and future clinical trials.
